# The Effect of *Lithospermum officinale*, Silver Sulfadiazine and Alpha Ointments in Healing of Burn Wound Injuries in Rat

**Published:** 2017-09

**Authors:** Zahra Mohtasham Amiri, Nader Tanideh, Anahita Seddighi, Maral Mokhtari, Masood Amini, Alborz Shakouri Partovi, Amir Manafi, Seyedeh Sara Hashemi, Davood Mehrabani

**Affiliations:** 1 Guilan Road Trauma Research Center, Department of Community Medicine, School of Medicine, Guilan University of Medical Sciences, Rasht, Iran;; 2 Stem Cell and Transgenic Technology Research Center, Shiraz University of Medical Sciences, Shiraz, Iran;; 3 Department of Pharmacology, School of Medicine, Shiraz University of Medical Sciences, Shiraz, Iran;; 4 Department of Genetics, Tehran Branch, Islamic Azad University, Tehran, Iran;; 5 Department of Pathology, School of Medicine, Shiraz University of Medical Sciences, Shiraz, Iran;; 6 Minimal Invasive Laparascopy Research Center, Shiraz University of Medical Sciences, Shiraz, Iran;; 7 Iran University of Medical Sciences, Tehran, Iran;; 8 Burn and Wound Healing Research Center, Shiraz University of Medical Sciences, Shiraz, Iran.

**Keywords:** Burn, *Lithospermum officinale*, Silver sulfadiazine, Alpha ointment, Wound healing, Rat

## Abstract

**BACKGROUND:**

Burn is the most devastating condition in emergency medicine leading to chronic disabilities. This study aimed to compare the effect of *Lithospermum officinale*, silver sulfadiazine and alpha ointments on healing of burn wounds in rat.

**METHODS:**

Ninety-five rats were divided into 5 groups. Group 1 just underwent burn injury, and groups 2-5 received alpha ointment, silver sulfadiazine (SSD), gel base and *L. officinale* extract, respectively. A hot plate was used for induction of a standard 3^rd^ degree burn wound. Burn wounds were macroscopically and microscopically evaluated on days 7^th^, 14^th ^and 21^st^ after burn induction.

**RESULTS:**

A decrease in the number of inflammatory cells was noted when *L. officinale* and SSD were applied while the most inflammatory response was seen after administration of alpha ointment. The number of macrophages alone decreased after burn injury, while the frequency was the most when *L. officinale *and alpha ointment were applied. Re-epithelialization, angiogenesis and formation of granulation tissue were the best in relation to *L. officinale *and alpha ointment while, the worst results belonged to burn injury group and SSD regarding granulation tissue formation. Considering histological assessment, the best results were observed for scoring of inflammation, re-epithelialization, angiogenesis, formation of granulation tissue and number of macrophage when *L. officinale *and alpha ointment were used after burn injury.

**CONCLUSION:**

It can be concluded that topical application of *L. officinale* as a non-toxic, inexpensive and easy to produce herbal can lead to a rapid epithelialization and wound healing and these findings can be added to the literature on burn wound healing.

## INTRODUCTION

In developed and developing countries, burn is still an emergency medicine resulting to psychological and physical scars and chronic disabilities.^1^^,^^2^ The outcome is dependent on the depth and size of the burn injury and the complications.^3^ The most existing problem is scarring in survived patients from the injury. Wound healing is consisted of inflammation, granulation, and remodeling of the tissue.^4^ Factors such as oxygen free radicals can contribute to delay the wound repair process. Therefore, early antioxidant therapy promotes the healing process and reduces the release of free oxygen radicals.^5^

The knowledge on wound healing and introduction of new technologies have opened a window to control the infections and antibiotic resistances in burn wounds.^6^ Silver sulfadiazine (SSD) has been introduced as a gold standard in burn therapy.^7^ Cho-Lee * et al.* realized that SSD can delay the wound-healing process with cytotoxic effects on the host cells^8^ and may be associated with resistance to many bacteria.^9^

In burn wound repair, herbal medicine was shown as treatment of choice^10^ such as honey with equal effects in healing of burn wounds in comparison to conventional therapies.^11^ For centuries, the traditional medicine were extensively used in wound healing of burned injuries.^11^^-^^14^ *Lithospermum officinale* is a herbal species of the genus Lithospermum. As a dietary supplement, it is used in oil form called borage oil (BO). *L. officinale* belongs to *Boraginaceae* family that was shown to have clinical efficacy in the suppression of inflammation in skin diseases.^15^^,^^16^ The major constituent of *Lithospermum* is oxidative metabolites of γ-linolenic acid (GLA), prostaglandin E1 (PGE1), and 15-hydroxyeicosatrienoic acid (15-HETrE), which have anti-inflammatory effects *in vitro*.^15^ *L. erythrorhizon*), another plant species of the *Boraginaceae* family native to East Asia, has been used traditionally in remedies for abnormal skin conditions, such as burns and inflammation.^17^

As *L. officinale* is one of the plants that was used for repairing burn from many years ago in north of Iran, this study was performed to determine the healing effect of this traditional medicine in healing of burn wounds in comparison to SSD and alpha burn ointments in rat as an experimental model.

## MATERIALS AND METHODS

During fall and winter of 2016, ninety five female Sprague-Dawley rats (180-220 g) were provided from Experimental and Comparative Medical Center of Shiraz University of Medical Sciences. Animal selection, care and the sacrifice procedures, and all experiments were the same and upon the instructions of Animal Care Committee of Iran Veterinary Organization. All experiments were conducted under aseptic conditions. The animals were kept one per cage under controlled environmental condition of 21±2ºC, 65–70% RH and a balanced diet with free access to food and water. The study was approved in Guilan University of Medical Scienses Ethics Committee.

*L. officinale* was provided from the Herbal Medicine Market, Rasht, Iran. The water extract of *L. officinale* was provided by decocting small pieces of *L. officinale* (100 g) with 500 ml of boiling distilled water for 3 h. The extract was filtered through Whatman no. 2 paper and lyophilized to prepare 15.0 g of water extract of *L. officinale*. Solid oil was later added to the extract. The animals were divided into 5 groups. Group 1 did not receive any medication and just underwent burn injury, Group 2 received alpha ointment (Sina Daru Co., Tehran, Iran), Group 3 was treated with silver sulfadiazine (SSD, Shafa Co, Tehran, Iran) ointment, Group 4 was cured with gel base and group 5 received *L. officinale*. 

Before induction of burn injuries, the animals were sedated by ketamine (15 mg/kg) and xylocain (1.1 mg/kg) intramuscularly and the back hairs were shaved and the skin was cleansed using povidone iodine and later by sterile water. A hot plate was used for induction of a standard 3^rd^ degree burn wound enrolling 20% total body surface area (TBSA).^18^ Burn wounds were assessed every 24 hours for any changes in wound’s appearance, the color, smell or any discharge and time of scar separation. Treatment measures were undertaken instantly and carried out twice per day. Animals with infected wounds or unstandard established wounds were excluded from the study and if died during the experiments were excluded from statistical analysis.

Burn wounds were macroscopically evaluated on days 7^th^, 14^th ^and 21^st^ after burn induction using digital camera. After 7^th^, 14^th ^and 21^st^ days, six animals were sacrificed with an overdose of anesthetics in each group and different time intervals. For histological studies, tissue samples were stained with hematoxylin and eosin. Histological assessment was done by scoring the presence of reepithelialization, angiogenesis, formation of granulation tissue, macrophages and inflammatory processes.^19^ The data were analyzed using SPSS software (version 11.5, Chicago, IL, USA) by ANOVA and PostHoc tests. A *p*<0.05 was considered statistically significant.

## Results

**Fig. 1:**
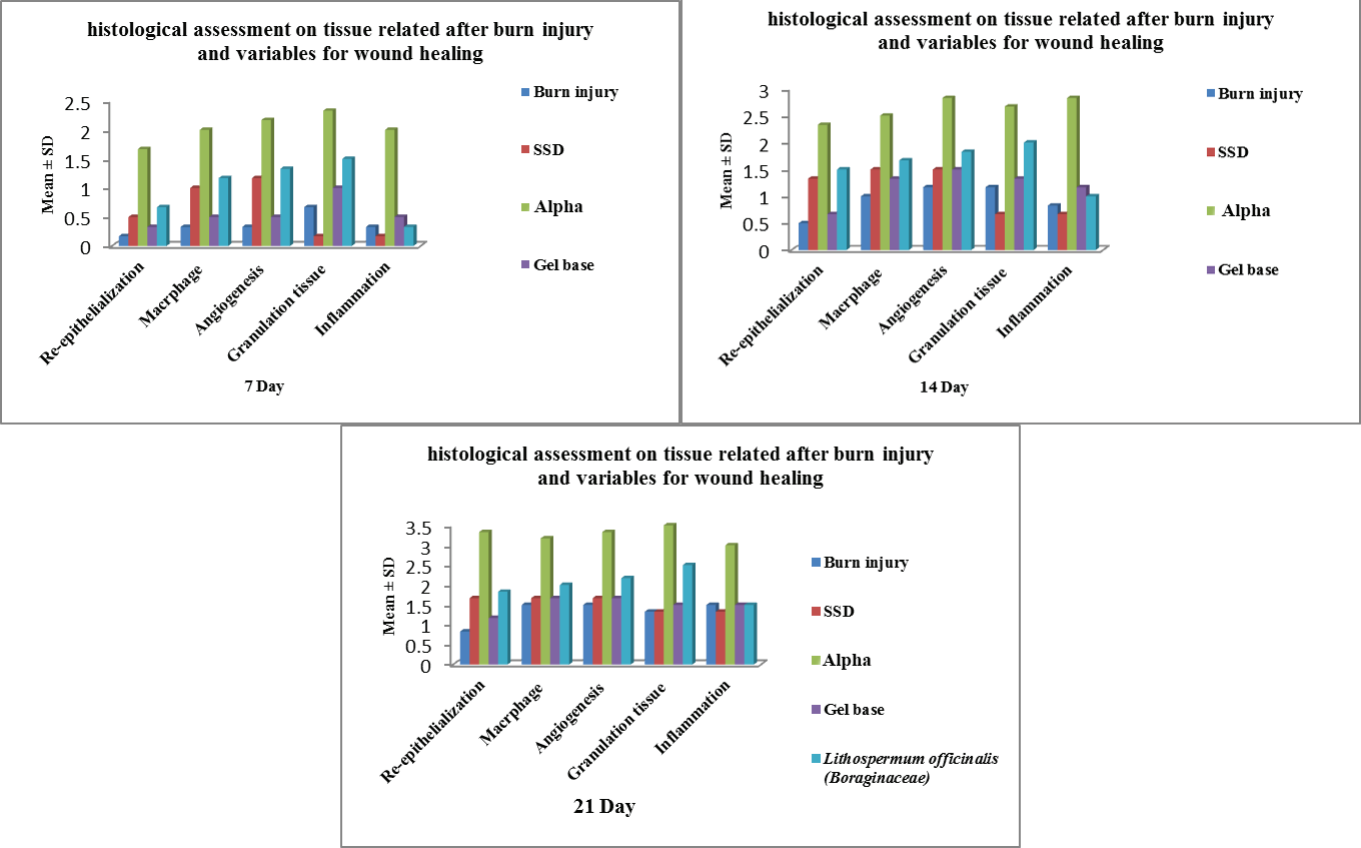
Comparison of histological assessments related to different days after burn injury for wound healing.

Macroscopically, the burn wound injury showed a decrease in size on days 7^th^, 14^th^ and 21^st^ in treatment groups without any significant difference. Figure 1 shows that on days 7^th^, 14^th^ and 21^st^, a decrease in the number of inflammatory cells including macrophages was noted when *L. officinale* and SSD were applied while the most inflammatory response was seen after administration of alpha ointment. The number of macrophages alone decreased after burn injury on days 7^th^, 14^th^ and 21^st^ while the frequency was the most when *L. officinale and *alpha ointment were applied. Re-epithelialization, angiogenesis and formation of granulation tissue were the best in relation to *L. officinale *and alpha ointment while on days 7^th^, 14^th^ and 21^st^, the worst results belonged to burn injury group and SSD regarding granulation tissue formation. 

**Fig. 2:**
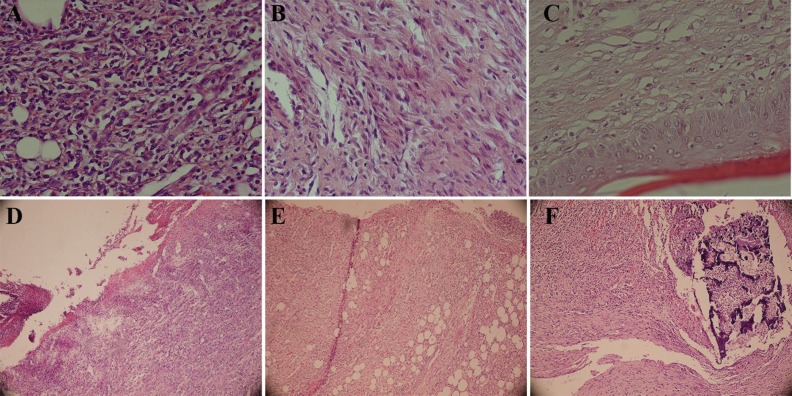
The effect of *L. officinale *in healing of burn wounds.** A **(*L. officinale*)**: **Severe inflammation, vascular proliferation, and no collagen and fibroblast proliferation**. B **(*L. officinale*)**: **Mild inflammation, marked fibroblast proliferation, and collagen deposition. **C **(*L. officinalis*)**: **Normal epidermis, mature granulation tissue, and collagen deposition. **D **(Control)**: ** Ulceration, marked inflammation, and vascular proliferation. **E **(SSD)**:** Ulceration, severe inflammation, and vascular proliferation. **F **(Alpha)**:** Marked inflammation, necrosis, and vascular proliferation

Regarding histological assessment of wound healing on days 7^th^, and 14^th^, the best results were observed for scoring of inflammation, re-epithelialization, angiogenesis, formation of granulation tissue and number of macrophage when *L. officinale *and alpha ointment were used after burn injury and on day 14^th^, the worse findings were visible in burn injury and the gel base groups (Table 1, Figure 2).

**Table 1:** Pathological studies on wound healing scoring in different groups

**Group**	**Day**	**Re-epithelialization**	**Granulation**	**Inflammatory**	**Macrophages**	**Angiogenesis**
Burn injury	7	0	0	0	0	0
Silver sulfadiazine	7	1	1	0	1	1
Alpha	7	2	2	2	2	2
Gel base	7	0	1	1	1	0
Lithospermum officinale (Boraginaceae)	7	1	2	0	1	1
Burn injury	14	1	1	1	1	1
Silver sulfadiazine	14	1	1	1	2	2
Alpha	14	2	3	3	3	3
Gel base	14	0	1	1	1	1
Lithospermum officinale (Boraginaceae)	14	2	3	1	2	2
Burn injury	21	1	1	1	1	1
Silver sulfadiazine	21	2	1	1	2	2
Alpha	21	3	3	3	3	3
Gel base	21	1	1	1	1	2
Lithospermum officinale (Boraginaceae)	21	2	3	2	2	2

Table 2 denotes to the difference between different groups for various parameters related to wound healing. Regarding inflammation, the best findings were found for *L. officinale *group when numbers of macrophages were also included in our counts and the worse results for alpha ointment group on days 7^th^, 14^th^ and 21^st^ after burn injury. No inflammation was noticed for *L. officinale*. Alpha ointment resulted into inflammation on days 7^th^, 14^th^ and 21^st^. Considering re-epithelialization, angiogenesis, granulation tissue formation, the best results were noted when *L. officinale *and alpha ointment were applied and the worse findings in burn injury group.

**Table 2:** Comparison of different groups for various parameters related to wound healing

**Group**	**Re-epithelialization** **(Mean±SD)**	**Macrophage** **(Mean±SD)**	**Angiogenesis** **(Mean±SD)**	**Granulation tissue** **(Mean±SD)**	**Inflammation** **(Mean±SD)**
Burn injury	0.5±0.13^a^	0.94±0.13^a^	1.00±0.12^a^	1.06±0.12^a^	0.89±0.13^a^
Silver sulfadiazine	1.67±0.13^b^	1.39±0.13^b^	1.44±0.12^b^	0.72±0.12^b^	0.72±0.13^a^
Alpha	2.34±0.13^c^	2.56±0.13^c^	2.78±0.12^c^	2.83±0.12^c^	2.60±0.13^b^
Gel base	0.72±0.13^a^	1.17±0.13^ab^	1.22±0.12^ab^	1.28±0.12^a^	1.06±0.13^a^
*Lithospermum officinale *	2.89±0.13^d^	1.61±0.13^b^	1.78±0.12^d^	2.00±0.12^d^	0.94±0.13^a^

Significant difference between different groups demonstrated with dissimilar superscript letters (*p*<0.05).

## DISCUSSION

Burn is a multifactorial trauma enrolling all body organs and impairing the patient’s physical, psychological, and social functioning, interpersonal relationships, aesthetic appearance, and all aspects of Health Related Quality of Life.^20^ In dermal burn injuries, the protective function of the skin that can be as a barrier to any infection is absent. Wound healing and tissue repair consist inflammatory processes, granulation tissue formation and remodeling of the tissue. Therefore, treatment of burn injury is based on healing process and preventing wound infection.^21^

It was shown that human amniotic membrane can be used successfully as a biological therapy in experimental third-degree burn injuries.^22^ Folk medicinal plants hav been used in wound healing successfully.^4^^,^^7^^,^^10^^,^^14^^,^^23^ *L. officinale* is among one of the most potent natural anti-inflammatory antioxidant materials that can be used in healing of burn wounds too. *L. officinale* was shown to have important ingredients such as phenolic acids, tannins, naphtoquinone with a traditional uses in France as gonadotropin antagonist, contraceptive.^24^ It also has the highest anti-inflammatory activity due to its ability to inhibit lipoxygenase activity.^24^ These propertis (inhibiting lipoxygenase activity) can explain the anti-inflammatory activity of *L. officinale* used in this study. Effective antioxidant activities were observed by comparison with two reference molecules, vitamin E and quercetin. These activities are possibly due to the presence of phenolic compounds that denote the healing effect of *L. officinale*.^24^ The whole plant was demonstrated to be diuretic and litholytic before.^25^ It has been introduced as an Iroquis indian drug used as a diuretic and pediatric aid based on its lithosenine and O3’-acetyllithosenine contents.^26^

In our study, we showed that based on scoring of inflammation, re-epithelialization, angiogenesis, formation of granulation tissue and number of macrophage, the best results for wound healing were observed when *L. officinale *was applied after burn injury. It can be concluded that topical application of *L. officinale* as a non-toxic, inexpensive and easy to produce hebal can lead to a rapid epithelialisation and wound healing and these findings can be added to the literature on burn wound healing.
